# Different Transcriptional Profiles of Human Monocyte-Derived Dendritic Cells Infected with Distinct Strains of *Mycobacterium tuberculosis* and *Mycobacterium bovis* Bacillus Calmette-Guérin

**DOI:** 10.1155/2011/741051

**Published:** 2011-03-22

**Authors:** Nunzia Sanarico, Alessia Colone, Manuela Grassi, Viviana Speranza, Daniela Giovannini, Antonio Ciaramella, Vittorio Colizzi, Francesca Mariani

**Affiliations:** ^1^Institute of Cell Biology and Neurobiology, National Research Council, Via del Fosso del Cavaliere, 100, 00133 Rome, Italy; ^2^National AIDS Centre, Istituto Superiore di Sanità, Viale Regina Elena 299, 00161 Rome, Italy; ^3^Department of Biology, University of Rome “Tor Vergata”, Via della Ricerca Scientifica, 00133 Rome, Italy; ^4^Department of Clinical and Behavioral Neurology, IRCCS Santa Lucia Foundation, Via Ardeatina 306, 00179 Rome, Italy

## Abstract

In order to analyze dendritic cells (DCs) activation following infection with different mycobacterial strains, we studied the expression profiles of 165 genes of human monocyte-derived DCs infected with H37Rv, a virulent *Mycobacterium tuberculosis *(MTB) laboratory strain, CMT97, a clinical MTB isolate, *Mycobacterium bovis* bacillus Calmette-Guérin (BCG), Aventis Pasteur, and BCG Japan, both employed as vaccine against tuberculosis. The analysis of the gene expression reveals that, despite a set of genes similarly modulated, DCs response resulted strain dependent. In particular, H37Rv significantly upregulated EBI3 expression compared with BCG Japan, while it was the only strain that failed to release a significant IL-10 amount. Of note, BCG Japan showed a marked increase in CCR7 and TNF-**α** expression regarding both MTB strains and it resulted the only strain failing in exponential intracellular growth. Our results suggest that DCs display the ability to elicit a tailored strain-specific immune response.

## 1. Introduction

How organisms respond appropriately to the wide variety of pathogens and antigens they encounter, and how pathogens can subvert the host immune response, has not been fully analyzed. The immune response to infectious agents involves a complex interaction of different cell types, and two defense arms have evolved to protect the host from microbial attack: a rapidly responding innate immune response to sequester and eliminate pathogens followed by a highly specific adaptive immune response. Dendritic cells (DCs) represent the bridge between the innate and adaptive immune response [1], and several studies support the hypothesis that DCs specifically strengthen the cellular immune response against mycobacterial infections [2, 3]. Even though the critical role of DCs in the initiation of immune response has been established [4], their involvement in *Mycobacterium tuberculosis* (MTB) infection is not completely characterized. Following pulmonary infection with MTB, DCs are activated rapidly to produce a specific pattern of chemokines and cytokines, key participants in the early immune response, and to express maturation markers that allow them to migrate to the draining lymph nodes. DCs become fully competent antigen presenting cells (APCs) and participate to the development of T helper 1 (Th-1) cells, required for the elimination of intracellular pathogens [4–6]. In particular, interleukin 12 (IL-12) produced by activated DCs induces Th-1 cells that, in turn, release interferon *γ* (IFN-*γ*) and tumour necrosis factor *α* (TNF-*α*). These cytokines activate intracellular microbicidal mechanism and initiate a protective granulomatous response [7]. The magnitude of host immune response depends, to a large extent, on the presence of costimulatory molecules and signaling lymphocytic activation molecules on DC surface, as well as local production of cytokines [8]. 

*Mycobacterium bovis* bacillus Calmette-Guérin (BCG) is a widely used vaccine against tuberculosis (TB) but comparative genetic analyses of BCG around the globe have revealed that each vaccine currently in use has different traits [9]. For decades, a number of factors have been considered responsible for the variable efficacy of BCG, including the type of strains used. In general, different kinds of stimuli result in differently activated DCs that induce qualitatively different T cell responses. Recently, it has been described that DCs are able to discriminate between phylogenetically diverse pathogens. In fact, the analysis of the DCs responses to *E. coli* and *C. albicans* showed that a unique number of genes, were regulated by each pathogen [10, 11]. However, the downstream target genes induced in DCs by the different BCG strains have not yet been fully determined. 

The importance of DCs in initiating an immune response against mycobacterial infections led us to investigate the activation induced on these APCs following stimulation with two widely employed and different BCG strains. The goal of the present study was to determine whether the strains divergences may influence their relative immunogenicity [12, 13], virulence [14, 15], and viability [16], factors that must be considered for the design and improvement of a vaccine against TB.

We also analyzed the DCs' response to the commonly used MTB virulent laboratory strain (H37Rv) and to an MTB clinical isolate (CMT97), previously reported to behave differently from H37Rv in human macrophages [17], in order to understand if the laboratory strain could be considered a real model of DCs-MTB interaction.

We aimed to understand whether the maturation reprogramming occurring on DCs, following infection with MTB H37Rv, MTB CMT97, BCG Aventis and BCG Japan, could be different as a consequence of the ability of DCs to discriminate between these mycobacterial strains. 

We used oligonucleotide macroarrays to characterize DCs gene expression profile and we found that although all infecting mycobacteria induced a core response, a strain-specific program emerged. The data obtained showed that BCG Japan was more effective than both MTB strains at inducing the expression of TNF-*α*, a gene involved in inflammation, as well as CCR7, responsible for DCs migration to lymph nodes. Furthermore, MTB H37Rv displayed, as compared to BCG Japan, an improved induction of EBI3, a IL-12p40-related polypeptide of IL-27 that may play a role in regulating cell-mediated immune response [18, 19], but, on the other hand, it resulted to be the only mycobacterial strain unable to promote a statistically significant IL-10 release as compared to uninfected cells. Moreover, IL-12 was significantly released only upon BCG Aventis infection. Finally, we also observed that BCG Japan displayed the lowest rate of intracellular replication as compared to BCG Aventis and both MTB strains.

## 2. Materials and Methods

### 2.1. Mycobacteria

Two pathogenic strains of MTB were chosen to infect DCs: the laboratory H37Rv strain (ATCC N° 27294) and the clinical isolate CMT97. CMT97 was isolated at the Monaldi Hospital, Naples, Italy, from a TB patient's sputum [20]. Both strains were transferred every two months to Sauton's medium, allowing them to grow as a layer on the medium surface. Mycobacterial layers were harvested every two months, spun down, and resuspended in phosphate-buffer saline (PBS). To get an homogeneous suspension, mycobacteria were placed in glass tubes and sonicated in a bath sonicator (UST; 20 kHz) at the maximum power of 50 W. Samples were than aliquoted and stored at −80°C. To titrate mycobacteria, few aliquots were thawed and grown on 7H10 Middlebrook plates (Becton Dickinson, Franklin Lakes, N.J.). The same frozen master batch was used for each infection experiment. As regards to BCG strains employed, we chose BCG Aventis Pasteur seed Merieux (BCG Aventis), derived from strain 1077 (Aventis Pasteur SA 2 Avenue Pont Pasteur, F-69007 Lyon), and 3-1-5, Japan BCG Laboratory Matsuyama, Kiyose-shi, Tokyo (BCG Japan). Both of them were supplied freeze-dried and intended for live inoculation. They were reconstituted according to the manufacturer's instructions.

### 2.2. Generation and Infection of DCs

Peripheral blood mononuclear cells (PBMC) were isolated, by Lympholyte-H (Cederlane, Canada) gradient centrifugation, from peripheral blood of healthy donors drawn from healthy volunteers. Monocytes were selected by anti-CD14-coated magnetic beads (MACS, Milteny Biothec, Germany). The purity was >98%, as verified by flow cytometry analysis. Cells were plated at 1.5 × 10^6^ cell/mL in RPMI-1640 medium (EuroClone, UK) supplemented with 10% heat-inactivated fetal bovine serum (FBS, HyClone, UT), L-Glutammin 2 mM, HEPES buffer 10 mM, sodium piruvate 1%, gentamicin 5 *μ*g/mL (all EuroClone, UK) adding GM-CSF 50 ng/mL and IL-4 10 ng/mL (all EuroClone, UK) to allow them to differentiate into DCs. After 5 days of differentiation, immature DCs derived from the same donor were exposed to MTB H37Rv, MTB CMT97, BCG Aventis Pasteur, and BCG Japan at a multiplicity of infection (MOI) of 1 bacterium per cell or left uninfected. Before infection bacilli were sonicated to disrupt small aggregates of bacteria. After 3 h of incubation at 37°C all DCs (both infected and uninfected ones) were harvested and centrifuged at 800 rpm for 10 min to spin down the DCs and leave extracellular bacteria in the supernatant. DCs were resuspended in fresh complete and, at the indicated time points, cells were collected and supernatants were stored at −80°C. For all the infections we chose a MOI of 1 since this condition did not result in rapid cell death and allowed us to culture DCs for at least 7 days after infection.

### 2.3. Flow Cytometry and Monoclonal Antibodies

The following monoclonal antibodies (mAbs), directly fluorochrome conjugated, were used for flow-activated cell sorting (FACS) analysis: anti-CD1a, anti-CD25, anti-CD80, anti-CD86, anti-CD83, anti-HLA-DR, anti-CD14, anti-CD11c, and anti-HLA-A,B,C. Negative controls were isotype-matched mAb (all from Becton Dickinson Biosciences). To determine surface cell phenotype, 24 h after infection, uninfected and infected DCs were washed in assay buffer (PBS, 0.5% BSA and 0.1% sodium azide), incubated with the above described mAbs for 15 min at +4°C, washed and then analyzed by flow cytometry.

### 2.4. Mycobacterial Enumeration by CFU Determination

Following infection, cells were washed and resuspended in fresh complete medium. At the indicated time points infected DCs were incubated for 30 min with 500 *μ*L of lysis buffer (PBS, 0.1% Saponin), diluted in PBS, 0.01% Tween 80, sonicated and plated as 50 *μ*L droplet on 7H10 plates in triplicates, at different dilutions. The CFU were checked after 21 days of dish culture at +37°C in a 5% CO_2_ incubator.

### 2.5. cDNA Arrays

Total RNA was extracted from both the uninfected DCs and the infected cells, 24 h after infection. RNA was extracted with 4 M Guanidine iso-thio-cyanate single-step method [21]. The extraction was performed on an RNase-free bench, in a separate room. Absorption spectroscopy was used to measure the purity and concentration of RNA with an A_260/280_ ratio of 2.0 indicating highly purified RNA. A total RNA sample (1.5 *μ*g) was reverse transcribed using the Ampolabellig Kit (Superarray Bioscience Corporation) according to the manufacturer's instruction in the presence of [*α*-^33^] P for the generation of radio-labeled cDNA probes. The probes were used to hybridize human Dendritic and Antigen Presenting Cell Gene Arrays (GEArray, S series; Supearray Bioscience Corporation) according to the manufacturer's instruction. Hybridization signals were detected by Phosphor-imager Thyphoon (Molecular Dynamics) and analyzed by Array Vision 7.0 software (Imaging Research Inc., Canada). Pathogen stimulations were repeated in three donors. Our data were expressed as normalized density (nDens) of each spot, corresponding to the density value of the spot minus background density and expressed as a multiple of the reference density value. The threshold of 0.001 was attributed to any value ≤0.001. We considered upregulated (+) and downregulated genes (−) only those ones that showed at least a twofold change in the level of RNA expression of the infected *versus* uninfected DCs in two of three independent experiments (fold change ≥2). Differences in the expression were calculated by dividing the gene nDens of infected cells by uninfected cells nDens.

### 2.6. Quantitative Real-Time Reverse Transcriptase-PCR (q-rt RT-PCR)

One *μ*g of total RNA, treated with DNAse I Amplification Grade (Invitrogen, Paisley, UK) was reverse transcribed using random examers and SuperScript III Reverse Transcriptase (Invitrogen, Paisley, UK), according to the manufacturer's instruction. For quantification of PCR products ABI PRISM 7000 SDS was used (Applied Biosystem, Foster City, USA). The RealMasterMix SYBR ROX (Eppendorf AG, Germany) was used to produce fluorescently labeled PCR products, and we monitored increasing fluorescence during repetitive cycling of the amplification reaction. For all primers, the following temperature cycling profile was used: 2 min a +50°C and 10 min at +95°C followed by 30 sec at Ta, and 1 min at +68°C for 40 cycles. Primer sequences are reported in Table 1. L34 were used as an internal control to normalize total RNA amounts.

### 2.7. Cytokine Determination by ELISA

DCs supernatants were collected at the end of the 24 h of culture and stored at −80°C. The amount of IL-10, IL-12 and TNF-*α* levels was evaluated by ELISA (Pierce Endogen, Woburn, MA) according to manufacturer's instruction.

### 2.8. Graphical, Statistical and Cytofluorimetric Analysis

The cytometric analysis was performed on a FACScalibur flow cytometer (BD Biosciences) and data were analyzed using CellQuest software (BD Biosciences). GraphPad Prism 4 (Graphpad software, San Diego, CA) was used for graphical and statistical analysis. Statistical significance was assessed by using analysis of variance (ANOVA), followed by Bonferroni's *post hoc* test; differences were considered significant at *P* < .05.

## 3. Results

### 3.1. Phenotype Analysis of Monocyte-Derived DCs Infected with Different Mycobacteria

**Figure 1:**
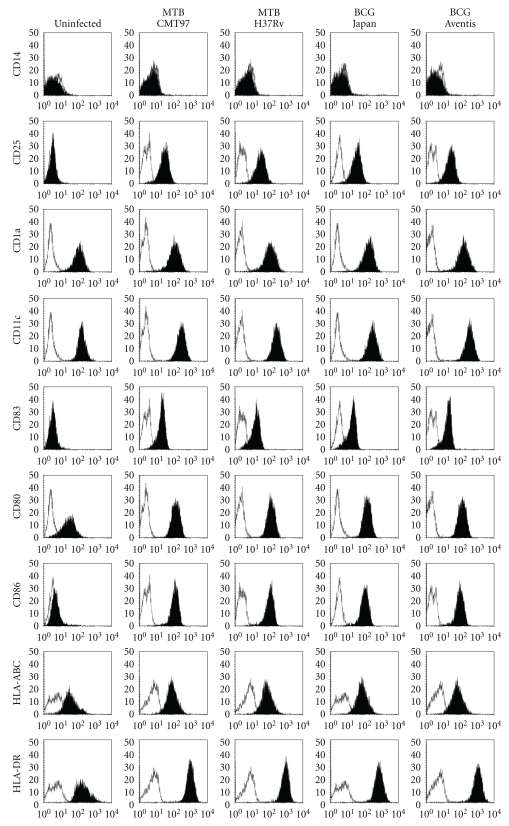
*Flow cytometry analysis of phenotype markers on DCs infected with different mycobacterial strains. *DCs, obtained from the same donor, were exposed to MTB H37Rv, MTB CMT97, BCG Aventis and BCG Japan for 3 h (MOI, 1) or left uninfected. Flow cytometric analysis of typical membrane molecule expression was performed 24 h after exposition to mycobacteria. Fluorescence histograms for each surface molecule (filled histograms) in comparison with isotype controls (empty histograms) are reported. Data are from a single donor, representative of 7, all with similar results.

In order to analyze DCs activation following infection with different mycobacterial strains, we evaluated the ability of MTB H37Rv, MTB CMT97, BCG Aventis and BCG Japan to induce the expression of maturation markers on DCs membrane. MTB H37Rv and MTB CMT97 were selected as both pathogenic: the first is the commonly used laboratory strain of MTB, while the second is a clinical isolate from a TB patient's sputum, displaying a peculiar clinical picture [20] and a specific ability to activate human mcrophages in comparison with MTB H37Rv [17]. We also chose two commonly employed vaccine strains: BCG Aventis and BCG Japan. DCs derived from the same donor were exposed to the different mycobacterial strains for 3 hours at a MOI of 1 bacteria per cell or left untreated, and, after 24 hours, infected and uninfected DCs were harvested, washed, and analyzed by flow cytometry for typical marker profiles. As shown in the representative histogram plots of Figure 1, MTB H37Rv and MTB CMT97 as well as BCG Aventis and BCG Japan were all efficient in stimulating DCs to undergo maturation when compared to uninfected DCs. This was assessed by the upregulation of activation markers such as HLA-ABC, HLA-DR, CD80, CD86, CD83, and CD25. The results obtained revealed that all the selected mycobacteria were found to induce phenotype maturation of DCs with a comparable efficacy.

### 3.2. Cell Vitality of DCs Following Stimulation with Different Mycobacterial Strains

**Figure 2:**
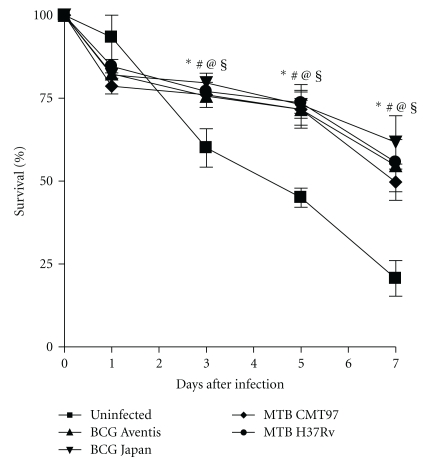
*Cell recovery of DCs following infection with different mycobacterial strains.* DCs infected or uninfected were enumerated by trypan blue at the indicated time points. Time 0 is referred to DCs before infection; following time points indicate days after infection. Data are shown as mean ± SEM from 3 independent experiments. Statistical significance was assessed by using two-way ANOVA. (Significant differences at 3 days after infection: *MTB H37Rv *versus* uninfected, *P* < .05; ^#^MTB CMT97 *versus* uninfected, *P* < .05; ^@^BCG Aventis *versus* uninfected, *P* < .05; **^§^**BCG Aventis *versus* uninfected, *P* < .05. significant differences at 5 and 7 days after infection: *P < .001; ^#^ *P* < .001; ^@^ *P* < .001; ^§^ *P* < .001.)

We further examined whether cell vitality, could be affected when comparing DC cells infection with the two pathogenic MTBs and the BCG vaccine strains. The viability of the DCs was evaluated, at different time points, in terms of percentage of living cells. In Figure 2 we showed that the cell yield, 1-day-culture after mycobacterial exposure, appeared diminished as an effect of the infection and that the reduction was comparable in all the infections. Recovery of infected DCs remained almost stable for the following 4 days, when alive cells started to decrease with a comparable trend. On the contrary, the number of alive, uninfected DCs remained almost stable for 1 day, then, as expected, unstimulated immature DCs showed a reduction in viability as compared to all the infected DCs at 3, 5, and 7 days. Taken together, these data indicate that all the mycobacterial strains did not produce cell death differently in 7-day-culture after infection.

### 3.3. Mycobacterial Intracellular Growth

**Figure 3:**
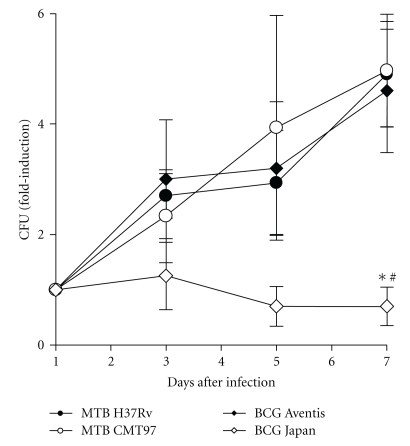
*Bacterial loads in DC infected with MTB H37Rv, MTB CMT97, BCG Aventis and BCG Japan.* DCs obtained from the same donor were exposed to different mycobacteria (MOI, 1). After 3 h, cells were washed then, part of them was checked for intracellular mycobacteria while the rest were resuspended in fresh medium and left in culture. Total bacterial load was assessed over the first 7 days and it is indicated as fold induction compared to the intracellular bacteria after 1 day from infection. The BCG Japan CFU were found significantly different at day 7 as compared to MTB H37Rv (*P < .01), MTB CMT97 (*P < .01) and BCG Aventis (^#^ *P* < .05). Data shown as mean ± SEM from 3 independent experiments performed in triplicates. Statistical significance was assessed by using two-way ANOVA.

To ascertain whether bacterial burden could be comparable when DCs were infected with different BCG and MTB strains, we monitored the number of intracellular mycobacteria over 7-day culture following infection. For the laboratory strain MTB H37Rv and the clinical isolate MTB CMT97, we found an increase in mycobacterial counts, and a comparable rate of growth was assessed in DCs infected with BCG Aventis (Figure 3). Specifically, enumerated colony forming units (CFU) displayed a 4-fold increase after seven days of infection. On the contrary, the intracellular ability to replicate turned out to be greatly impaired when counting the intracellular BCG Japan CFU. In fact, the mycobacterial number remained almost steady over all the monitored period and resulted statistically reduced at the 7th day of infection, as compared to the other three mycobacteria. These results suggest that DCs differently contain the intracellular growth of the mycobacteria strains analyzed.

### 3.4. Gene Expression Profile of DCs Infected with Different MTB and BCG Strains

To test whether the mycobacterial strains we selected could differently modulate the overall gene expression of DCs, we analyzed the expression of 165 genes involved in DC activation and maturation using macroarrays. We analyzed and compared the expression profile of DCs exposed for 3 h to MTB H37Rv, MTB CMT97, BCG Aventis and BCG Japan, 1 day-culture after the infection. The gene modulation, showed in Table 2, was confirmed in at least two of three independent assays, where each assay was performed on DCs derived from the same donor. In general, 30 genes were detected as upregulated (+) (fold change ≥2 in infected DCs *versus* uninfected DCs), corresponding to 18% of the 165 spotted onto the membrane (Table 2(a)), and 33 genes out of 165 (20%) were detected as downregulated (−) (Table 2(b)). In more detail, only 17 out of 30 genes proved to be comparably upregulated in all infected DCs while the other 13 genes resulted induced upon the encounter of DCs with some of the four mycobacteria. Analogous results were obtained when analyzing the downregulated genes. In particular, despite the fact that all the different mycobacteria decreased the expression of 25 gene, other 8 genes resulted repressed only following some of the infection performed. As expected, among the commonly upregulated genes we found cytokines involved in proinflammatory immune response such as IL-1*β*, IL-6, IL-12 and TNF-*α*, the regulatory IL-10 and chemokines and their receptors able to trigger DCs migration to lymph nodes (ADAM19, CCR7 and CCL20). On the other hand, we found, as commonly downregulated, genes that are substantially involved in antigen capture, loading and presentation as DC-SIGN, that is known to be responsible for DCs-T interaction. In this group we also found IL-18, which plays an important role in enhancing IFN-*γ* production by T cells [22], which proved to be preferentially downregulated in BCG Japan-infected DCs. In order to exactly appreciate gene expression and modulation we showed, in Table 3, nDens of all genes differentially regulated, following mycobacteria exposure. Moreover, we indicated nDens of 2 genes induced and 2 genes repressed in all infection performed and nDens of all genes discussed above (IL-1*β*, IL-6, IL-12, TNF-*α*, IL-10, ADAM19, CCR7, CCL20 and IL-18). In conclusion, the macroarray analysis suggests that different mycobacterial strains such as MTB H37Rv, MTB CMT97, BCG Aventis and BCG Japan induce, on DCs, a pathogen-specific response, both in upregulated and downregulated genes, in the face of a common set of gene that appear to be similarly modulated.

### 3.5. Expression of Selected Genes by q-rt RT-PCR

**Figure 4:**
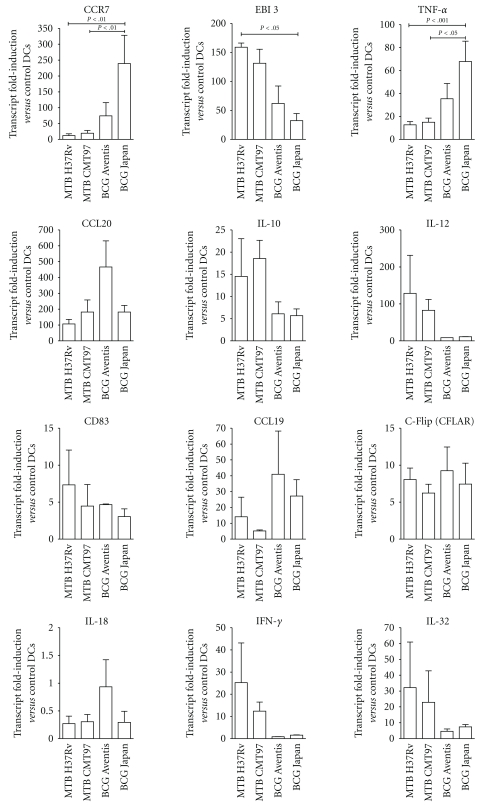
*Analysis of RNA modulation by q-rt RT-PCR*. Total RNA from DCs exposed for 3 h to MTB H37Rv, MTB CMT97, BCG Aventis and BCG Japan was assayed for gene expression by q-rt RT-PCR, 1-day culture after the infections. Gene expression was normalized to L34 and fold change was calculated with respect to uninfected DCs. Data are from 3 individual experiments and are expressed as mean ± SEM. The presence of significant differences in gene expression between DCs was calculated by a one-way ANOVA.

The mRNA levels of 12 selected genes were further analyzed by quantitative real-time PCR using gene specific primers (Figure 4). Six of these genes (CD83, CCL20, CCR7, IL-10, IL-12, and TNF-*α*) proved to be similarly upregulated in response to all the four mycobacterial strains but, due to their important role in the modulation of immune response, we further analyzed their expression in order to appreciate some possible quantitative difference. We also chose CCL19, c-FLIP (CFLAR), EBI3, and IL-18, which appeared differently modulated following the four infections, in order to verify such dissimilarity. For its well-documented pivotal role in MTB host response, we also decided to include in this analysis IFN-*γ*, even if it was present in the array and did not show any significant modulation in DCs following infection. We also included in q-rt RT-PCR analysis IL-32, absent in macroarray, since this has been recently shown to be involved in antitubercular immunity [23], Moreover, it has been described that mycobacterial species such as *M. tuberculosis* or *M. bovis* BCG are potent stimuli for the production of the proinflammatory cytokine IL-32 and its production is dependent on endogenous IFN-*γ* [24]. The result obtained confirmed the upregulation of CCL20, IL-10, IL-12, previously observed with the macroarray assay, in DCs exposed to the MTB as well as the BCG strains. Also the similar upregulation of CD83, already described at the protein level in Figure 1, was confirmed. Interestingly, the q-rt RT-PCR pointed out a significantly higher mRNA transcript of CCR7 and TNF-*α* in BCG Japan-infected-DCs, as compared to MTB H37Rv- and MTB CMT97-infected DCs (*P* < .01 and *P* < .05, resp.). 

Concerning CCL19, c-FLIP and IL-18, whose expression resulted different in DCs after the encounter with the two MTB and two BCG strains (Table 2), when we reanalyzed the mRNA levels by quantitative real-time PCR, the overall expression of these genes was comparable among the different infections although a certain degree of variability was observed. 

In agreement with macroarray analysis, EBI3 expression resulted clearly induced in MTB H37Rv- and MTB CMT97-infected DCs, but a lower RNA induction was observed also in BCG infected DCs. In addition, a statistical analysis of EBI3 modulation underlined a consistent upregulation of this gene only in MTB H37Rv infected-DCs as compared to BCG Japan infected-DCs. In line with the modulation of EBI3, the upregulation of IFN-*γ* and IL-32 resulted to be stronger in MTB H37Rv and MTB CMT97 infected-DCs but no statistically significant differences can be described. 

Collectively, these findings, consistent with the macroarray data, allow us to describe a different modulation of the selected genes, thanks to the higher sensitivity of the quantitative real-time PCR as compared to the macroarray assay. Furthermore these data suggest that the maturation of DCs, following the infection with different MTB and BCG strains, may result in a different modulation of some genes importantly involved in the response against mycobacteria.

### 3.6. Release of IL-10, IL-12 and TNF-*α* from Infected and Uninfected DCs

**Figure 5:**
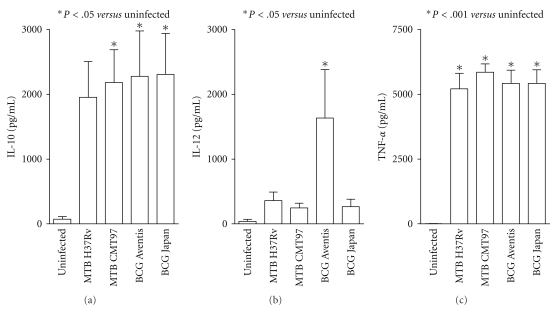
*Evaluation of IL-10, IL-12 and *TNF-*α released from infected DCs.* Cell supernatants derived from DCs uninfected or exposed to different mycobacteria strains as described before were collected at the end of the 24 h of culture. IL-10, IL-12 and TNF-*α* were measured by ELISA. Data are represented as mean ± SEM from 3 independent experiments. The presence of significant differences in the amount of cytokine released in the supernatant was calculated by a one-way ANOVA.

DCs have the unique capacity to stimulate naïve T lymphocytes driving these cells into a distinct class of effector cells. To investigate whether the infection with different mycobacterial strains could result in a different cytokine release from DCs, cell supernatants were collected 24 h after infection and the presence of IL-10, IL-12 and TNF-*α* was quantified by ELISA. We chose IL-10 and IL-12 since they are crucial for driving Th-1/Th-2 response and TNF-*α*, that already proved differently modulate at the RNA level, for its well-documented protective role against MTB and for its ability to mature DCs [25, 26]. As shown in Figure 5, a significantly higher release of IL-10 was observed from both BCG-infected and MTB CMT-infected DCs as compared to uninfected cells even though DCs exposed to MTB H37Rv also release a discrete amount of this regulatory cytokine.

Concerning IL-12, a consistent and significant production was found only in BCG Aventis-infected DCs while, in the other 3 infections, only a mild increase in the level of IL-12 was observed as compared to control DCs. Finally, the TNF-*α* released and accumulated in the supernatant for 24 h after infection resulted high and similar in all the infections, as compared to uninfected DCs (*P* < .001).

## 4. Discussion

MTB is an extremely well adapted pathogen that coexisted with the human host for thousands of years and during this period it has learnt how to modulate potentially protective host responses, to ensure its own survival. H37Rv is the currently used MTB laboratory strain and, considering how important it is to have a good *in vitro* model, it could be necessary to assess if H37Rv is able to induce a host response at least comparable to a clinical isolate. In fact, it has been clearly shown that different MTB clinical isolates have distinct effects and produce a different response that depends on their specific virulence [27, 28]. We decided to include in our study the strain CMT97, a clinical isolate previously characterized in infected cells of bronchial lavage fluid and in human infected macrophages [17, 20]. 

In parallel, BCG has emerged as a vaccine that changed from the original Pasteur strain into a range of strains that immunized many people around the world, with variable results. In this context, a characterization of a strain-specific host response and in particular a critical analysis and comparison between two widely used BCG strains could lead to a more rational approach towards the improvement of the BCG vaccine. In the present study, we investigated the DCs response to mycobacterial infections and we chose two different MTB and BCG strains: the laboratory strain H37Rv, CMT79, a clinical isolate from a TB patient, and two vaccine frequently used nowadays (BCG Aventis and BCG Japan). Several studies have been published dealing with the DCs response to different mycobacteria [10, 27, 28] but this is the first time that two different BCGs, an MTB clinical isolate and a laboratory MTB strain are compared simultaneously for the activation induced on human monocyte-derived DCs coming from the same donor. 

First, we compared the selected mycobacteria for their ability to replicate inside DCs and, interestingly, it emerged that only BCG Japan was unable to enlarge the intracellular bacterial population in infected DCs. This suggests, in accordance with *in vivo* previous studies [29], that during the initial phase of infection with MTB H37Rv, MTB CMT97 and BCG Aventis, the growth of the bacterial population could be accompanied by an increment of the number of infected cells, which implies a high rate of cell-to-cell spread of the mycobacteria, while the cell-to-cell spread outcome following the infection with BCG Japan may possibly occur to a lesser extent. Of note, although DCs contained differently the intracellular growth of BCG Japan compared to the other three mycobacteria, cell expression of activation markers as well as cell recovery proved to be comparable unrelated to the infecting mycobacteria. This supports the fact that DCs differently contain the intracellular growth of the mycobacteria considered, while cells similarly survive after all the infections performed.

It's important to keep in mind that, since 1921, the *in vitro* attenuation of *M. bovis* gave rise to a large number of BCG daughter strains, which have been classified as “early strains” and “later strains”. The BCG Japan is an “early strain” while BCG Aventis Pasteur belongs to the “later strains”, obtained before the loss of the RD14 region [9, 30]. Considering this, it is not surprising that different BCG strains could have such a different adaptive feature and that the differences existing between the BCG daughter strains may influence the activation of infected DCs. To explore globally the host gene expression differences we performed a DCs comparative *in vitro* transcriptome analysis across the two MTB and two BCG strains. The analysis of 165 genes involved in cell maturation and activation showed that 18% of these proved to be upregulated and 20% were detected as downregulated. More in detail, 17 out of 30 genes resulted to be comparably upregulated in all infected DCs and resulted to be involved in proinflammatory immune response (Il-1*β*, IL-6, IL-12 and TNF-*α*), immune regulation and migration (IL-10, ADAM19, CCR7 and CCL20) showing that all mycobacteria were able to elicit the expression of many genes already described as upregulated in common pathogen response [10]. The other 13 genes resulted differently induced and among these we chose CCL-19, C-Flip and EBI3 to be further investigated by q-rt RT-PCR. 

Accordingly with the macroarray analysis, EBI3 resulted preferentially induced in the DCs infected with MTB virulent species than BCG a-virulent strains. Interestingly, when comparing the gene-fold induction across the four mycobacteria, H37Rv displayed a significantly higher EBI3 transcription as compared to BCG Japan. EBI3 is a subunit of IL-27 that, together with IL-12, is involved in driving commitment of naïve T cells to a Th-1 phenotype [31, 32]. Intriguingly, it has been recently published that neutralization of IL-27 reduced, even if modestly, viable MTB recovered from macrophages [33]. In this scenario, the lower EBI3 induction in BCG Japan-infected DCs might be involved in BCG Japan failing to grow exponentially in DCs.

The comparison, by q-rt RT-PCR, of gene-fold induction across the four mycobacteria also revealed that the proinflammatory TNF-*α* and the migratory chemokine receptor CCR7 were strongly induced in BCG Japan-infected DCs as compared to cells infected with both MTB strains. This suggests that BCG Japan might prove particularly efficient at promoting DCs migration into T cell-enriched areas of lymphoid tissue, where DCs are able to present Ag-derived peptides, associated with either class I or class II MHC molecules to naïve CD4 and CD8 T cells, respectively [34]. Moreover, previous studies have shown that TNF-*α* is fundamental in granuloma formation and maintenance and also affects cell migration upon MTB infection, since it influences the expression of adhesion molecules as well as chemokines and chemokine receptors such as MIP-1*α*, MIP-1*β*, RANTES, and CCR5 [35–38]. When we assayed the release of TNF-*α* from supernatants, we observed that this proinflammatory cytokine was found to be abundantly released in all infected DCs. This result strongly supports the evidence that initial interaction of MTB and BCG induces proinflammatory cytokine production [27, 39].

As previously shown [40], we observed a consistent release of IL-10 from BCG-infected DCs even if these cells resulted to be well activated following mycobacteria exposure, as assessed by the upregulation of maturation markers. Also, MTB CMT97-infected DCs produced significantly higher levels of IL-10 as compared to uninfected cells while, MTB H37Rv induced the release of a discrete but not significantly amount of this cytokine. When we measured the release in the supernatant of IL-12, a cytokine pivotal in directing the polarization of immune response, we observed that despite the fact that a RNA upregulation was described in all MTB and BCG infections, only BCG Aventis induced a significant IL-12 production, while BCG Japan and both MTB strains turned out to be a stimulus insufficient for the induction of IL-12 releasing DCs. The data obtained, at a protein level, confirm the previously reported ability of MTB H37Rv and different MTB clinical isolates to suppress the secretion of IL-12 by monocyte-derived DCs [27] and, also suggest another possible difference in the immune activation induced by the two different BCG analysed. 

Of note, even if there is an evident discrepancy between RNA analysis and protein detection from supernatants, it should be remembered that several experimental studies have shown that mRNA changes do not necessarily correlate with changes in the corresponding proteins, which are the ultimate determinants of cellular function [41]. The invalid assumption of the one-to-one correlation between the ratios of protein levels and the corresponding mRNAs [42, 43] suggests that the relation between transcription and translation, and consequently between mRNA and protein, is complex. Moreover, qRT-PCR shows what is happening in a particular moment, while the ELISA assay we performed gives us the total amount of protein, released in the course of 24 hours following infection.

In conclusion, the importance of DCs in initiating immune response gave reason to investigate if these cells could discriminate between a clinical and a laboratory MTB strain and also if DCs could sense differently two distinct BCG strains. The analysis of the individual response showed that DCs exhibit stimulus-specific maturation and activation. In fact, besides a shared core reprogramming, DCs are able to modulate the expression of exclusive genes, proving that these immune regulating cells are able not only to discriminate between phylogenetically distinct pathogens [10] but also, elicit a specific response as respect to diverse MTB and BCG strains. The results obtained describe the contribution to pathogen-host interaction of strictly correlated mycobacterial strains. In particular, our data indicate that the specific differential response to MTB H37Rv and MTB CMT97 mimics the previously reported different cellular response to distinct clinical MTB isolate, dependently to their specific virulence [27, 28] supporting the validity of MTB H37Rv as an *in vitro* model for mycobacterial-DCs interaction. On the other side, the strain-specific modulation observed in response to BCG Aventis and BCG Japan as well as the different ability of these mycobacteria to growth in infected DCs lead to important physiological consequence that must be considered and further studies of BCG-regulated genes may thus enhance our understanding of DCs maturation and provide future indication for the design and improvement of a vaccine against tuberculosis.

## Figures and Tables

**Table 1:** Primer sequences used in the q-rt RT-PCR.

Gene	Sense	Antisense	Annealing temp. (°C)
L34	5′-GGCCCTGCTGCATGTTTCTT-3′	5′-GTCCCGAACCCCTGGTAATAGA-3′	64
EBI3	5′-AGAGCACATCATCAAGCCCGAC-3′	5′-TCCCTGACGCTTGTAAGCGCATC-3′	64
CCR7	5′-AAAAGCGTGCTGGTGGTGGC-3′	5′-ATGATAGGGAGGAACCAGGC-3′	64
TNF-*α*	5′-AGGCGGTGCTTGTTCCTCA-3′	5′-GTTCGAGAAGATGATCTGACTGCC-3′	60
CCL19	5′-CCAATGATGCTGAAGACTGC-3′	5′-CTGGATGATGCGTTCTACCC-3′	62
CCL20	5′-TGAAGGCTGTGACATCAATGC-3′	5′-TGTTTTGGATTTGCGCACAC-3′	60
IL-12	5′-GCTGCTGAGGAGAGTCGTCCC-3′	5′-CCAGCTGACCTCGACCTGCC-3′	62
IL-10	5′-AGGCGCATGTGAACTCCCT-3′	5′-CACGGTCTTGCTCTTGTTTT-3′	64
IFN-*γ*	5′-GGCTGTTACTGCCAGGACCCCATATGT-3′	5′-GATGCTCTTGCACCTCGAAACAGCCAT-3′	64
IL-32	5′-GACATGAAGAAGCTGAAGGCC-3′	5′-ATCTGTTGCCTCGGCACCG-3′	62
IL-18	5′-GACAATTGCATCAACTTTGTGG-3′	5′-ATAGAGGCCGATTTCCTTGG-3′	62
c-FLIP	5′-TTCATGGGAGATTCATGCCC-3′	5′-AAGAGGCTGCTGTCCTCCA-3′	60
CD83	5′-AGGTTCCCTACACGGTCTCC-3′	5′-TTGAAGCTGGTAGTGTTTCG-3′	60

**Table:** (a)

Gene	MTB H37Rv	MTB CMT97	BCG Aventis	BCG Japan	Common name	Classification	Function
ADAM19	+	+	+	+		Metalloproteinase	Migration/Inflammation
BASP1	+	+	+	+	CAP-23, NAP-2	Cell surface protein	Prot-prot interaction
CCL5	+	+	+	+	RANTES	Chemokine ligand	Migration
CCL20	+	+	+	+	MIP-3*α*	Chemokine ligand	Inflammation
CCL22	+	+	+	+	MDC	Chemokine ligand	Inflammation
CCR7	+	+	+	+		Chemokine receptor	Migration
CD83	+	+	+	+		Cell surface protein	Ag presentation
IL-1*β*	+	+	+	+		Cytokine	Inflammation
IL-6	+	+	+	+		Cytokine	Inflammation
IL-10	+	+	+	+		Cytokine	Immune regulation
IL-12p40	+	+	+	+		Cytokine	T cell stimulation
LY75	+	+	+	+	DEC-205	Cell surface receptor	Ag presentation
NFKB1	+	+	+	+		Trascriptional factor	Signal transduction
SOD2	+	+	+	+		Mytocondrial protein	Oxidative stress
TAP2	+	+	+	+		Ag transporter 2	Ag presentation
TLR2	+	+	+	+		Toll-like receptor	Pathogen assoc. receptor
TNF-*α*	+	+	+	+		Cytokine	Inflammation
ADAR	+			+		Adenosine deaminasi	RNA modification
CCL3	+	+	+		MIP-1*α*	Chemokine ligand	Inflammation
CCL19			+	+		Cell surface receptor	Migration
CD80				+		Cell surface protein	Ag presentation
CD86		+	+	+		Cell surface protein	Ag presentation
CFLAR			+	+	C-Flip	Apoptosis regulator	Apoptosis inhibitor
CRF			+			C1q related factor	
EBI3	+	+				Secreted glicoprotein	Immune-regulation
IL-1a		+	+	+		Cytokine	Inflammation
LAMP3	+	+		+	CD63	Lysosomal associated protein	Ag capture
MT2A		+	+			Metallothionein	Oxidative stress
PLAUR		+	+		CD87	Cell surface receptor	Migration
TNFRSF6			+	+	CD95, FAS	Cell surface receptor	Maturation

**Table:** (b)

Gene	MTB H37Rv	MTB CMT97	BCG Aventis	BCG Japan	Common name	Classification	Function
ARHGDIB	−	−	−	−		RhoGDP dissociation inhibitor 2	Cell motility and adhesion
CD1A	−	−	−	−		MHC I like protein	Ag presentation
CD1B	−	−	−	−		MHC I like protein	Ag presentation
CD1C	−	−	−	−		MHC I like protein	Ag presentation
CD36	−	−	−	−		Cell surface protein	Ag capture
CD68	−	−	−	−		Cell surface protein	Ag capture
CD74	−	−	−	−	Invariant chain	MHC II assoc. protein	Ag loading
CD209	−	−	−	−	DC-SIGN	C-type lectin receptor	T/DCs interaction
CLECSF12	−	−	−	−	DECTIN-1	Cell surface receptor	Pattern recog. receptor
CLECSF6	−	−	−	−	DCIR	C-type lectin receptor	Ag capture
CST3	−	−	−	−		Proteinase inhibitor	
CXCL16	−	−	−	−		Chemokine	T cell stimulation
FCER1A	−	−	−	−		Fc receptor	Inflammation
FCER2	−	−	−	−		Fc receptor	Inflammation
GIP3	−	−	−	−		IFN-*α* inducible protein	
HLA-DMA	−	−	−	−		MHC II accessory protein	Ag loading
HLA-DMB	−	−	−	−		MHC II accessory protein	Ag loading
IFI16	−	−	−	−		IFN-*γ* inducible protein	Cell cycle regulator
IFITM3	−	−	−	−		IFN inducible protein	Immune response
ITGB2	−	−	−	−		Integrin	Migration
LANGERIN	−	−	−	−		Cell surface protein	Ag capture
LIPA	−	−	−	−	Lipase A	Acid lipase	Lipidic metabolism
MX1	−	−	−	−		IFN-*α* inducible p78	Antiviral response
RNASE6	−	−	−	−		Ribonuclease	Ag presentation
TLR4	−	−	−	−		Toll-like receptor	Pathogen assoc. receptor
CSF1R	−	−		−	CD115	Cell surface receptor	M-CSF receptor
DCP1B	−			−		Decapping enzyme hDcp1b	RNA degradation
DCSTAMP	−		−			Cell trasmemb. protein	
GBP3				−		Guanilate binding protein	
GIP2	−			−		IFN-*α* inducible protein	
IL-18				−		Cytokine	Inflammation
PFN1				−			
TLR6	−			−		Toll-like receptor	Pathogen assoc. receptor

Upregulated (Table 2(a)) and downregulated (Table 2(b)) genes of DCs exposed for 3 h to MTB H37Rv, MTB CMT97, BCG Aventis and BCG Japan as compared to uninfected DCs were assayed by macroarray 1-day culture after infections. For genes to be referred as upregulated or downregulated we considered only those that showed at least a twofold change in the level of RNA as compared to uninfected DCs expression, in at least 2 independent experiments of the 3 performed.

**Table:** (a)

Gene	MTB H37Rv	MTB CMT97	BCG Aventis	BCG Japan	Uninfected DCs
ADAM19	0.3; 0.4; 1.5	0.3; 0.2; 1.8	0.2; 0.7; 0.1	0.3; 0.6; 1	0.2; 0.07; 0.5
CCL20	0.4; 1; 0.8	0.4; 2.5; 1.4	0.9; 2.3; 0.8	0.5; 0.8; 1.1	0.04; 0.001; 0.4
CCL22	8.5; 1.8; 11.1	6.9; 3.7; 12.4	4.3; 1.8; 12.1	8.1; 1.5; 9.8	0.3; 0.5; 0.2
CCR7	3.1; 0.001; 1.7	3.2, 0.9; 1.9	0.5; 1.8; 1.6	2.4; 0.9; 2.2	0.1; 0.2; 0.05
IL-1*β*	2.1; 2.9; 14.4	1.7; 10.5; 19.0	0.3; 6.5; 10.8	1.9; 4.8; 14	0.09; 0.9; 0.8
IL-6	0.5; 0.02; 2.9	0.1; 0.4; 3.4	0.001; 0.6; 2.3	0.1; 0.3; 3.2	0.01; 0.01; 0.1
IL-10	1.8; 3.7; 6.6	1.0; 1.2; 8.9	1.2; 1.3; 5.8	1.0; 0.9; 4	0.4; 0.3; 4.4
IL-12p40	0.6; 0.3; 0.9	0.6; 0.7; 0.7	0.7; 0.1; 1.0	0.4; 0.01; 1.5	0.1; 0.1; 0.004
SOD2	0.8; 1.6; 5.5	0.8; 3.4; 7.6	0.9; 1.5; 3.7	0.5; 1.5; 5.4	0.1; 0.7; 0.7
ADAR	0.4; 0.2; 0.2	0.2; 0.1; 0.1	0.3; 0.1; 0.1	0.4; 0.3; 0.2	0.2; 0.1; 0.1
CCL3	4.1; 7.0; 11.0	3.6; 6.5; 5.6	7.0; 4.6; 10.8	2.5; 3.7; 1.2	1.7; 3.4; 0.6
CCL19	0.1; 0.1; 0.001	0.3; 0.007; 0.001	0.2; 0.3; 0.4	0.2; 0.1; 0.5	0.001; 0.1; 0.001
CD80	3.3; 0.4; 0.2	3.2; 0.5; 0.2	3.9; 0.9; 0.2	5.8; 0.7; 0.9	2.1; 0.3; 0.2
CD86	0.3; 0.001; 0.1	0.2; 0.02; 0.4	0.1; 0.1; 0.4	0.2; 0.01; 0.4	0.05; 0.02; 0.2
CFLAR	0.4; 0.5; 0.3	0.5; 0.5; 0.3	1.1; 1.3; 0.2	0.8; 0.8; 0.4	0.5; 0.3; 0.2
CRF	0.3; 0.3; 0.001	0.3; 0.1; 0.01	1.1; 0.2; 0.001	0.1; 0.02; 0.001	0.3; 0.1; 0.001
EBI3	0.1; 0.2; 0.5	0.1; 0.001; 0.7	0.01; 0.001; 0.2	0.01; 0.001; 0.3	0.01; 0.001; 0.008
IL-1*α*	0.02; 0.01; 0.4	0.1; 0.6; 0.5	0.3; 0.6; 0.3	0.4; 0.3; 0.9	0.02; 0.01; 0.001
LAMP3	5.1; 24.0, 1.6	10.6; 31.3; 2.1	13.4; 12.9; 2.3	21.8; 26.0; 1.7	9.3; 11.8; 0.7
MT2A	0.2; 0.1; 11.1	0.4; 0.5; 11.5	0.4; 0.2; 7.1	0.08; 0.1; 8.4	0.2; 0.2; 0.1
PLAUR	0.06; 0.1; 1.5	0.1; 0.7; 0.7	0.2; 0.4; 1.2	0.06; 0.1; 1.2	0.1; 0.1; 0.1
TNFRSF6	0.6; 0.1; 0.001	0.7; 0.006; 0.004	1.6; 0.4; 0.001	1.4; 0.2; 0.001	0.6; 0.001; 0.001

**Table:** (b)

Gene	MTB H37Rv	MTB CMT97	BCG Aventis	BCG Japan	Uninfected DCs
CD1A	0.1; 0.2; 0.001	0.1; 0.001; 0.02	0.06; 0.001; 0.05	0.2; 0.001; 0.001	5.2; 0.3; 1.1
CD1B	0.04; 0.4; 0.001	0.05; 0.001; 0.02	0.05; 0.001; 0.06	0.008; 0.001; 0.07	1.3; 0.001; 0.7
CD209	0.05, 0.1; 0.08	0.02; 0.03; 0.001	0.04; 0.001; 0.03	0.01; 0.2; 0.03	0.3; 0.1; 0.4
CSF1R	0.05; 0.4; 0.1	0.1; 0.5; 0.001	0.5; 0.2; 0.04	0.1; 0.1; 0.3	0.3; 0.2; 0.5
DCP1B	0.2; 0.001; 0.04	0.3; 0.001; 0.1	0.3; 0.1; 0.07	0.1; 0.07; 0.01	0.4; 0.1; 0.1
DCSTAMP	1.3; 0.001; 0.001	1.6; 0.001; 0.3	0.2; 0.05; 0.02	2.1; 0.01; 0.001	3.0; 0.1; 0.05
GBP3	0.3; 0.4; 0.1	0.08; 0.5; 0.03	0.1; 0.8; 0.05	0.1; 0.3; 0.04	0.1; 0.7; 0.1
GIP2	0.05; 0.001; 0.9	0.08; 0.3; 0.6	0.07; 0.4; 0.4	0.001; 0.1; 0.7	0.3; 0.3; 0.7
IL-18	0.1; 1.9; 0.2	0.1; 1.6; 0.2	1.1; 1.8; 0.2	0.1; 0.6; 0.2	0.2; 2.7; 0.2
PFN1	0.9; 0.5; 3.1	0.6; 0.4; 5.0	0.7; 0.6; 2.3	0.7; 0.2; 3.5	0.8; 0.5; 8.5
TLR6	0.05; 0.09; 0.001	0.08; 0.3; 0.03	0.1; 0.3; 0.001	0.001; 0.1; 0.001	1.4; 0.3; 0.02

RNA expression of infected and uninfected DCs of three independent experiments. Values are reported as nDens.
